# Pingwei San Ameliorates Spleen Deficiency-Induced Diarrhea through Intestinal Barrier Protection and Gut Microbiota Modulation

**DOI:** 10.3390/antiox12051122

**Published:** 2023-05-19

**Authors:** Yimeng Fan, Qingyu Zhao, Yuanyuan Wei, Huiru Wang, Yu Ga, Yannan Zhang, Zhihui Hao

**Affiliations:** 1 National Key Laboratory of Veterinary Public Health Security, College of Veterinary Medicine, China Agricultura University, Beijing 100193, China; b20223050425@cau.edu.cn (Y.F.); zhaoqingyu@cau.edu.cn (Q.Z.); b20203050389@cau.edu.cn (Y.W.); s20213050856@cau.edu.cn (H.W.); s20203050825@cau.edu.cn (Y.G.); b20193050423@cau.edu.cn (Y.Z.); 2 Key Biology Laboratory of Chinese Veterinary Medicine, Ministry of Agriculture and Rural Affairs, Beijing 100193, China; 3 National Center of Technology Innovation for Medicinal function of Food, National Food and Strategic Reserves Administration, Beijing 100193, China

**Keywords:** pingwei san, spleen deficiency diarrhea, gut microbiota, intestinal mucosal barrier

## Abstract

Pingwei San (PWS) has been used for more than a thousand years as a traditional Chinese medicine prescription for treating spleen-deficiency diarrhea (SDD). Nevertheless, the exact mechanism by which it exerts its antidiarrheal effects remains unclear. The objective of this investigation was to explore the antidiarrheal efficacy of PWS and its mechanism of action in SDD induced by Rhubarb. To this end, UHPLC-MS/MS was used to identify the chemical composition of PWS, while the body weight, fecal moisture content, and colon pathological alterations were used to evaluate the effects of PWS on the Rhubarb-induced rat model of SDD. Additionally, quantitative polymerase chain reaction (qPCR) and immunohistochemistry were employed to assess the expression of inflammatory factors, aquaporins (AQPs), and tight junction markers in the colon tissues. Furthermore, 16S rRNA was utilized to determine the impact of PWS on the intestinal flora of SDD rats. The findings revealed that PWS increased body weight, reduced fecal water content, and decreased inflammatory cell infiltration in the colon. It also promoted the expression of AQPs and tight junction markers and prevented the loss of colonic cup cells in SDD rats. In addition, PWS significantly increased the abundance of *Prevotellaceae*, *Eubacterium_ruminantium_group*, and *Tuzzerella*, while decreasing the abundance of *Ruminococcus* and *Frisingicoccus* in the feces of SDD rats. The LEfSe analysis revealed that *Prevotella*, *Eubacterium_ruminantium_group*, and *Pantoea* were relatively enriched in the PWS group. Overall, the findings of this study indicate that PWS exerted a therapeutic effect on Rhubarb-induced SDD in rats by both protecting the intestinal barrier and modulating the imbalanced intestinal microbiota.

## 1. Introduction

Spleen-deficiency diarrhea (SDD) is a prevalent gastrointestinal condition in traditional Chinese medicine (TCM) characterized by diarrhea as the primary symptom. In TCM, Qi is believed to be a vital force that drives various biological activities in the human body, including the functioning of organs and the transport of nutrients. For many gastrointestinal disorders, it is believed that Qi may become diminished or depleted [1]. The primary causes of SDD are believed to be the consumption of Qi, spleen insufficiency, stomach disharmony, and endophytic dampness, which cause intestinal conductive dysfunction [2]. Due to the weakened spleen’s inability to transport and the weakened stomach’s failure to digest and process food, the water and food are retained, affecting the ascent of the spleen yang, which eventually collapses, resulting in diarrhea. SDD is characterized by diarrhea, which is the primary symptom, along with secondary manifestations such as weight loss, chills, and fatigue. The syndrome also includes symptoms similar to those of inflammatory bowel disease (IBD) and ulcerative colitis (UC) [3].

Extensive studies in traditional Chinese medicine (TCM) have focused on the pathogenesis of SDD [3,4]. Modern medical research has further revealed that SDD is closely associated with digestive dysfunction in the gastrointestinal tract, inflammation in the intestine, reduced immune function [5], and imbalances in water metabolism [6]. The intestine is the primary organ involved in diarrhea, and its proper function relies on a healthy intestinal barrier, appropriate microbiota levels, and controlled inflammation [7]. The intestinal microbiome plays a vital role in preserving the integrity of the intestinal mucosal barrier, and any disturbance in this equilibrium can result in a dysfunctional intestinal mucosal barrier, which in turn, can lead to several diseases, including IBD and irritable bowel syndrome [8,9]. Disruption of the intestinal barrier can contribute to increased permeability, inflammation, and other pathological changes, which can lead to diarrhea [10]. Therefore, maintaining the integrity of the intestinal barrier and a balanced microbial environment is crucial in treating diarrhea [11]. The combination of tight junction proteins and the cellular immune system serves as a critical defense against external factors in healthy individuals [12]. Previous studies have shown a reduction in ZO-1 and Occludin expression in cases of diarrhea. As the significance of the gut microbiota and intestinal barrier in the development of diarrhea is increasingly acknowledged [13,14], it is crucial to explore the mechanisms by which TCM can regulate these factors to achieve its therapeutic effects.

Treatment of SDD often involves strengthening the spleen and supplementing Qi. Various TCM prescriptions, including Fuzi Lizhong decoction [15], Lingzhu decoction [16], Shenling Baizhu San (SLG) [4,17], and Pingwei San (PWS) [18], are used to treat SDD. PWS, which was first documented in the Prescriptions of Taiping Benevolent Dispensary [19], consists of four herbs, including *Atractylodes lancea* (Thunb.) DC., *Magnolia officinalis* Rehd. et Wils., *Citrus reticulata* Blanco, and *Glycyrrhiza uralensis* Fisch. From the perspective of TCM, PWS is believed to address damp-cold stagnation in the spleen and stomach by eliminating dampness, enhancing the spleen’s transportation function, promoting the movement of Qi, and harmonizing the stomach. The characteristics and benefits of PWS have been extensively described in Chinese literature, with documented abilities to combat infantile diarrhea, chronic atrophic gastritis, and chronic superficial gastritis [18]. In this study, we used SLG as a positive control, which includes *Atractylodes macrocephala* Koidz, *Smilax glabra* Roxb, *Coix lacryma-jobi var.* ma-yuen (Rom.Caill.) Stapf, *Panax ginseng* C.A.Mey, etc., and has been shown to effectively treat SDD [17]. As research has progressed, it has become increasingly clear that changes in the gut microbiota play a crucial role in the development and progression of SDD, leading to the occurrence of diarrhea [20]. *Atractylodes lancea* (Thunb.) DC., as the main herb of PWS, has been reported to protect against SDD by regulating the intestinal inflammatory response and gut microbiota [21]. 

Nevertheless, the specific mechanisms through which PWS exerts its antidiarrheal effects are not yet well understood. Therefore, this study aims to examine the healing potential of PWS in treating SDD and elucidate the mechanisms involved, with a particular emphasis on the modulation of gut microbiota and maintenance of intestinal barrier function.

## 2. Materials and Methods

### 2.1. Preparation of PWS, Rhubarb, and Shenling Baizhu San (SLG)

PWS was obtained from Tongrentang (Beijing, China), the components of PWS consist of *Atractylodes lancea* (Thunb.) DC. (80 g), *Magnolia officinalis* Rehd. et Wils. (60 g), *Citrus reticulata* Blanco (40 g), and *Glycyrrhiza uralensis* Fisch (20 g) (200 g in total). First, ingredients were soaked in 8 times of distilled water for 30 min and then extracted twice for 40 min each time with equal volumes of distilled water (25 °C). The resulting extracts were combined, boiled at 70 °C, and concentrated to 200 mL.

Rhubarb was cut into pieces, soaked in water, and kept in an oven at a temperature of 60–80 °C for the whole night. The resulting juice was squeezed out, filtered, and steamed to make a liquid solution with a concentration of 2 g/mL. The positive control used in this study, SLG, was procured from Tongrentang (Beijing) and made into a suspension by adding the required amount of water to achieve a concentration of 250 mg/mL [3].

### 2.2. Analysis of the Main Components of PWS

The ACQUITY UPLC HSS T3 column (2.1 × 100 mm, 1.8 μm particle size, 35 °C) was used with an injection volume of 10 μL, and the mobile phase consisted of aqueous formic acid (0.1%, *v*/*v*) and acetonitrile (0.1% formic acid) with a flow rate of 0.3 mL/min. The elution gradient was performed in several steps, ranging from 0–100% B over 45 min, followed by 15 min of re-equilibration. The gradient elution procedure is shown in Table 1. The Q Exactive Orbitrap was used to obtain mass spectral data in Full MS-ddMS2 mode while scanning in both positive and negative ion mode over the *m*/*z* range of 100–1200. The MS1 resolution was set to 70,000 and MS2 resolution to 17,500. The data were analyzed with Compound Discoverer 3.3 software, and compounds were identified using both the local and online MzCloud databases.

### 2.3. Animal Models and Treatment

Male Sprague Dawley (SD) rats that were 8 weeks old and of SPF grade were procured from SPF Biotechnology Co., Ltd (Beijing, China). (SYXK 2019-0010). The rats were acclimated for one week at 25 °C, 45–50% humidity, and a 12 h light/dark cycle. The animal trials were executed in accordance with the regulations specified by the Institutional Animal Care and Use Committee of China Agricultural University (AW010203202-2-3).

A total of 35 rats were randomly divided into five groups: control, SDD, PWS-L (1.5 g/kg), PWS-H (3 g/kg) [22], and SLG (dose settings refer to previous studies) [3]. All groups except the control group were administered rhubarb-derived liquid at a dose of 20 mL/kg twice per day (morning and evening) at an 8 h interval for a total of 7 days. Then, the corresponding drugs were administered to each group for 5 days. The control group was administered an equivalent amount of physiological saline. At the end of the trial, the colons were extracted, washed with phosphate-buffered saline (PBS), and assessed. Blood samples were collected from the rats and preserved at −80 °C. The colons and feces were cryopreserved at −80 °C until further analysis.

### 2.4. Record of Body Weights and Fecal Moisture Content

The weight of the rats and the moisture content of their feces were recorded. On Day 3 and Day 8, fecal samples were collected and weighed. Afterward, the feces were dried for 24 h at 60 °C and weighed again.

$$\mathrm{Fecal}\;\mathrm{moisture}\;\mathrm{content}\;(\%)=\frac{weight\;\;before\;\;drying-weight\;\;after\;\;drying}{weight\;\;before\;\;drying}\;\times \;100\%$$

### 2.5. D-Xylose Absorption and Excretion Assay

The method used for the assay was adapted from previous studies [23,24]. The rats were fasted for 4 h and given 2 mL of 3% d-xylose (Macklin, Shanghai, China) orally. One hour after the intervention, blood samples were collected from the post-ocular vein, and the serum was isolated through centrifugation. The concentration of d-xylose in the serum was determined using the d-xylose Assay Kit (Jiancheng, Nanjing, China) following the manufacturer’s instructions.

### 2.6. Histopathological Observation and Alcian Blue-Periodic Acid Schiff (AB-PAS) Staining

The colon tissue was fixed in a solution of 4% paraformaldehyde, embedded in paraffin, sliced into 4-μm sections, and stained with hematoxylin and eosin (H & E) stain (Solarbio, Beijing, China). The stained tissues were examined using a microscope (Olympus, Tokyo, Japan) and the pathological changes were observed through a light microscope (Nikon Eclipse CI, Tokyo, Japan).

Samples were stained with Alcian Blue solution (Solarbio, Beijing, China) and treated with periodic acid and Schiff’s reagent for AB-PAS staining. Hematoxylin was used to stain the nuclei lightly. Acid alcohol was used to differentiate the samples, which were then washed with Scott’s tap water. Finally, the samples were dehydrated, cleared, and mounted on glass slides with coverslips. The number of goblet cells was counted under a light microscope.

### 2.7. Immunohistochemistry (IHC)

The endogenous peroxidase activity was removed from the paraffin sections by treating them with 3% hydrogen peroxide. Afterward, the sections were blocked with 5% BSA and incubated with primary antibodies, including AQP3 (1:100, Bioss, Beijing, China), AQP4 (1:100, Bioss, Beijing, China), and AQP8 (1:100, Bioss, Beijing, China), at 4 °C for 10 h. After that, the sections were rinsed thrice with TBS and treated with the secondary antibody at 37 °C. The results were captured using a light microscope (DS-Ri2, Nikon, Japan), and the findings were quantified by utilizing Image-Pro Plus 6.0 software.

### 2.8. Enzyme-Linked Immunosorbent Assay (ELISA)

In order to obtain the 10% colon homogenate, 100 milligrams of colon tissue were combined with 900 microliters of normal saline, and subsequently subjected to centrifugation at 3500× *g* for a duration of 15 min. Inflammatory markers such as TNF-α, IL-1β, and IL-6 proteins were measured using ELISA kits for each corresponding protein. 

### 2.9. Measurement for T-AOC, GSH-Px, and Superoxide Dismutase (SOD) Activities in the Colon, Serum of Rats

About 50 mg of colon tissue samples were taken and mixed with 0.5 mL of chilled Tris buffer. The mixture was then homogenized and centrifuged at 3000× *g* for 15 min at 4 °C, and the resulting supernatant was collected. To determine the levels of T-AOC (A015-2-1), GSH-Px (A005-1-2), and SOD (A001-3) activities in both the colon and serum, commercially available kits (Jiancheng, Nanjing, China) were used as per the manufacturer’s instructions.

### 2.10. The Permeability Test of Intestinal Mucosal Barrier

To evaluate the permeability of the intestinal epithelial barrier, rats were subjected to a fluorescein isothiocyanate-labeled dextran permeability test after being anesthetized for 30 min. A section of the ileum, measuring 10 cm in length, was tied off at both ends of the abdominal wall’s midline. Subsequently, a solution of 1 mL 4 KD dextran (10 mg/mL, Sigma-Aldrich, St. Louis, MO, USA) labeled with fluorescein isothiocyanate was injected into the intestine. After 30 min, the arterial blood was collected. The upper plasma was analyzed using a fluorescence enzyme labeling instrument (PerkinElmer, Waltham, MA, USA) at 480 nm emission light to assess changes in the intestinal mucosal barrier’s permeability.

Concurrently, colon tissue was isolated and quickly frozen on dry ice after embedding in Tissue-Tek O.C.T compound (Sakura, Tokyo, Japan). A frozen section with a thickness of 10 μm was prepared using the CM1950 cryostat (Leica Biosystem, Wetzlar, Germany) and then observed under a fluorescence microscope (Olympus CKX53, Tokyo, Japan) for image acquisition.

### 2.11. Quantitative Reverse-Transcription (qRT)-PCR for the Gene Expression

RNA was isolated from colon tissues using RNA extraction (Promega, Shanghai, China) and subjected to reverse transcription using the GoScript™ Reverse Transcription System (Promega, Shanghai, China). To quantitatively analyze the genes, (qRT)-PCR was performed with the primer sequences for the related genes specified in Table 2. The 2^−ΔΔCT^ method was used to determine the relative expression of the target genes.

### 2.12. Determination of Fecal Flora Diversity

The methodology utilized in this study was based on our previous work [25]. In brief, fecal samples were subjected to DNA extraction and assessed for quality using spectrophotometry. PCR was then conducted (KAPA HiFi HotStart ReadyMix PCR Kit) with DNA polymerase and a diluted genomic DNA template. Specific primers (341F: 5′-CCTAYGGGRBGCASCAG-3′ and 806R: 5′-GGACTACNNGGGTATCTAAT-3′) were used to amplify the V3–V4 region. The Sibiocore platform was utilized to evaluate operational taxonomic unit clusters, and species classification analyses, as well as α- and β-diversity. The sequencing data was analyzed using QIIME 1.80, and the VSEARCH (v2.3.4) software was used to cluster sequences with 97% similarity into operational taxonomic units (OTUs). The GreenGene database was used for annotation purposes. For alpha diversity analysis, the mothur (v1.31.2) software was used. To assess the similarity of microbial relative abundance, PCA and PCOA analyses were conducted.

### 2.13. Statistical Analysis

The data was expressed using the mean and standard deviation (SD) and analyzed with the SPSS (Version 26; IBM, Armonk, NY, USA). Statistical significance was determined by conducting one-way analysis of variance (ANOVA), followed by SNK post-test where *p* < 0.05 was considered statistically significant. The graphics were created using GraphPad Prism (Version 9; La Jolla, CA, USA).

## 3. Results

### 3.1. Chemical Profile of PWS

**Figure 1:**
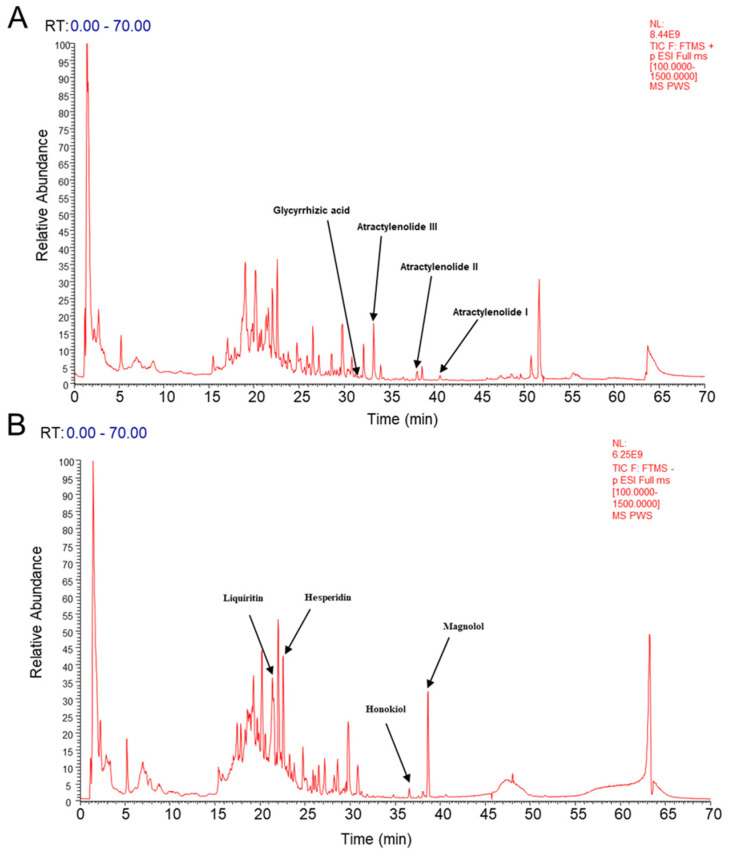
UHPLC–MS fingerprint chromatogram of QJD. (**A**) Positive mode. (**B**) Negative mode.

To determine the primary chemical constituents of PWS, UHPLC-MS/MS was utilized to analyze the relevant signals. A total of 287 chemical compounds were detected in PWS and were categorized into five major groups: flavonoids, terpenoids, coumarins, alkaloids, and glycosides (Table S1). The total ion chromatogram of PWS was displayed in Figure 1A,B. Through comparison with the reference sample’s retention time, as well as ultraviolet and mass spectrometry, Atractylenolide III, Atractylenolide II, Atractylenolide I, Magnolol, Honokiol, Hesperidin, Liquiritin, and Glycyrrhizic acid were identified (Table 3). 

### 3.2. PWS Ameliorates SDD Symptoms in Rats

**Figure 2:**
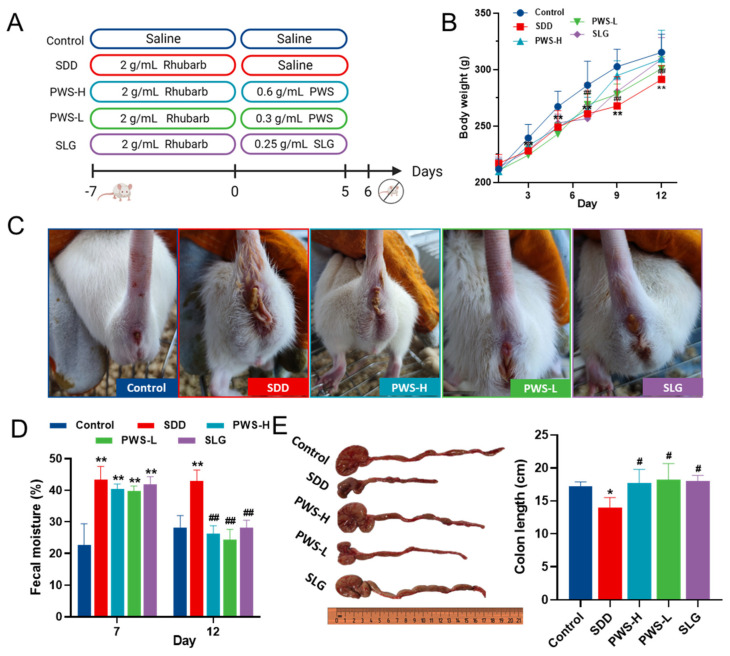
PWS attenuates diarrhea in SDD rats. (**A**) The experimental design of this study. (**B**) The changes in the body weight of the rats. (**C**) The incidence of diarrhea. (**D**) Fecal moisture content. (**E**) Representative images of the rat’s colon and the colon length. Control: control group; SDD: spleen-deficiency diarrhea group; PWS-H: Pingwei San high-dose group; PWS-L: Pingwei San low-dose group; SLG: Shenling Baizhu San group. The significance levels for *p*-values are indicated as follows: * *p* < 0.05, ** *p* < 0.01 vs. control group, # *p* < 0.05, ## *p* < 0.01 vs. DSS group.

The animal experiment scheme and design are illustrated in Figure 2A. Compared to the control group, the SDD group showed a slower rate of weight gain; however, all rats gained weight faster after PWS administration (Figure 2B). The administration of rhubarb caused diarrhea in rats and increased their fecal water content. In contrast, rats treated with PWS had significantly lower fecal water content than those in the SDD group (Figure 2C,D). Rhubarb treatment resulted in reduced colon length, which was markedly improved by different doses of PWS (Figure 2E). 

### 3.3. PWS Suppresses Inflammation and Inflammation-Induced Oxidation in SDD Rats

**Figure 3:**
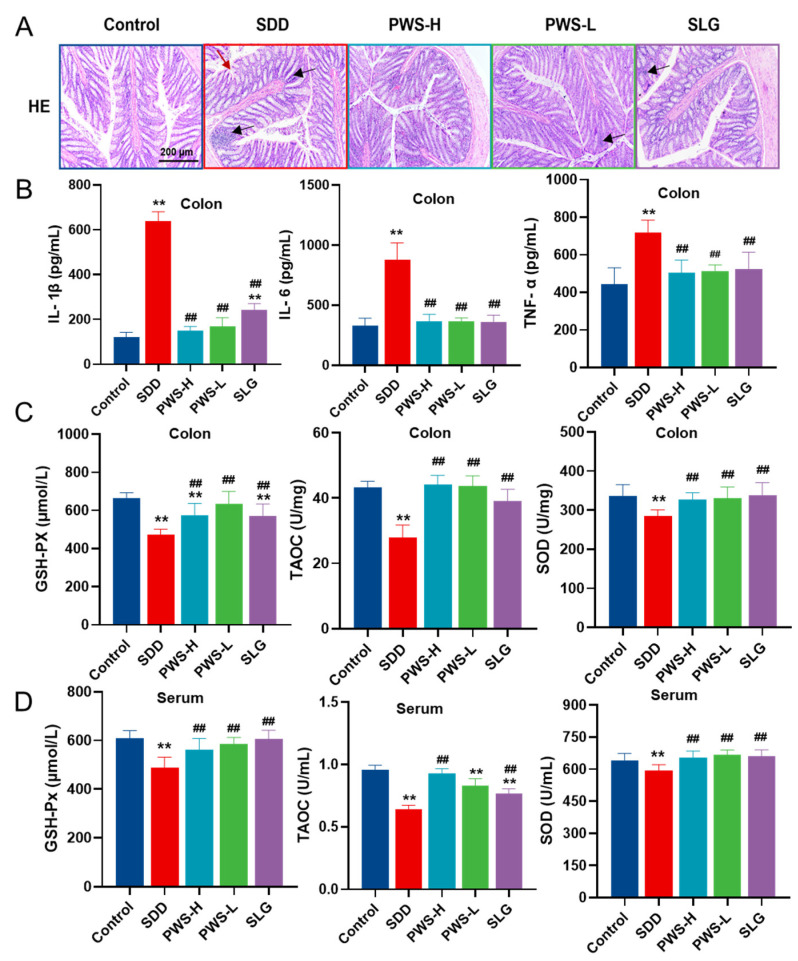
PWS suppresses inflammation and inflammation-induced oxidation in SDD rats. (**A**) Representative histopathological changes in the colon tissues of rats. The black arrows point to inflammatory cells and the red arrows point to disordered cell arrangement. (**B**) The levels of IL-1β, IL-6, and TNF-α in the colon tissues. (**C**) The levels of TAOC, GSH-Px, and SOD in the colon tissues of rats were measured. (**D**) The levels of TAOC, GSH-Px, and SOD in the serum of rats were measured. Control: control group; SDD: spleen-deficiency diarrhea group; PWS-H: Pingwei San high-dose group; PWS-L: Pingwei San low-dose group; SLG: Shenling Baizhu San group. The significance levels for *p*-values are indicated as follows: ** *p* < 0.01 vs. control group, ## *p* < 0.01 vs. DSS group.

The SDD group exhibited inflammation infiltration (black arrows) and disordered cell arrangement (red arrows) in colon tissues compared to the control group. However, treatment with PWS had a protective effect on SDD-induced diarrhea in rats, resulting in normal mucosal shape and intact epithelium in the intestine and colon tissues (Figure 3A). The impact of PWS on the expression of colonic cytokines (TNF-α, IL-6, and IL-1β) in SDD rats was analyzed to examine its effects on pathological inflammation. As indicated in Figure 3B, the expression levels of TNF-α, IL-6, and IL-1β were significantly increased in the model group, while PWS administration significantly decreased the expression levels (*p* < 0.01). To determine the oxidative stress biomarkers, TAOC levels and the activities of GSH-Px and SOD in the colon and serum were assessed. The results showed that SDD diarrhea significantly reduced the levels of TAOC and the activities of GSH-Px and SOD. Nevertheless, PWS notably alleviated the oxidative stress damage caused by SDD in the colon tissues (*p* < 0.01) (Figure 3C,D). The levels of TAOC, GSH-Px, and SOD activities were significantly increased in the PWS-treated group compared to the control group, indicating that PWS inhibited inflammation and inflammation-induced oxidation.

### 3.4. PWS Restored Flora Diversity to Curb SDD

**Figure 4:**
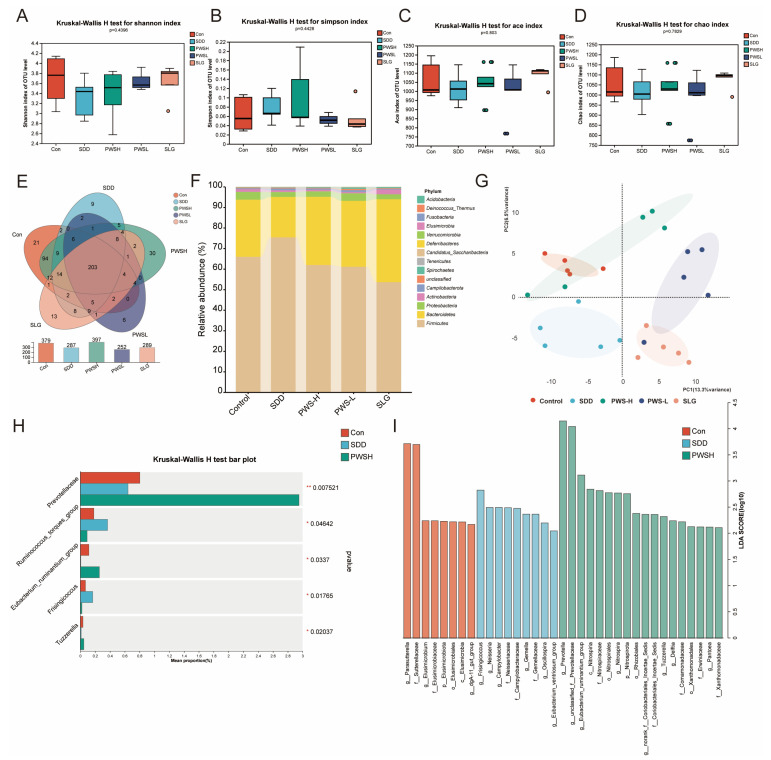
(**A**) Shannon index. (**B**) Simpson index. (**C**) Ace index. (**D**) Chao index. (**E**) Venn diagram of species in the five groups. (**F**) Relative abundance of phylum. (**G**) Beta diversity. (**H**) Relative abundance of genus. (**I**) LEfSe. Control: control group; SDD: spleen-deficiency diarrhea group; PWS-H: Pingwei San high-dose group; PWS-L: Pingwei San low-dose group; SLG: Shenling Baizhu San group. * *p* < 0.05, ** *p* < 0.01.

The use of natural active products can influence changes in the intestinal flora, which may contribute to the development of SDD. SDD and PWS were found to alter the alpha diversity (Shannon, Simpson, Chao, and ace) of the fecal flora (Figure 4A–D). The Venn diagram was used to identify common or unique taxa among the different groups, and a total of 379 OTUs in the control group, 287 ZOTUs in the SDD group, 397 OTUs in the PWS-H group, 252 OTUs in the PWS-L group, and 289 OTUs in the SLG group were detected (Figure 4E). The species bar chart was used to show the changes in species composition and proportion among groups. SDD reduced the relative abundance of *Firmicutes* and increased that of *Bacteroidetes*, while PWS reversed the changes (Figure 4F). The NMDS analysis and plots of PCA indicated that the control, SDD, and PWS groups had distinct community compositions of fecal flora, and PWS was able to normalize the bacterial community after SDD (Figure 4G). Figure 4H displays the genus-level relative abundance of intestinal flora in rats. In comparison to the control group, the SDD group exhibited an increase in the relative abundance of *Ruminococcus* and *Frisingicoccus* and a decrease in the relative abundance of *Prevotellaceae*, *Eubacterium_ruminantium_group*, and *Tuzzerella* (*p* < 0.01). Treatment with PWS restored the relative abundance of related bacterial genera in SDD rats to normal levels. We identified a significant difference in the dominance of bacterial communities among the five groups by using lLEfSe. According to the analysis results, *Facklamia* and *Oscillospira* were the key bacterial types that caused an imbalance in intestinal microflora in the SDD group. However, *Prevotella*, *Eubacterium_ruminantium_group*, and *Pantoea* were relatively enriched in the PWS group (Figure 4I).

### 3.5. PWS Increases the Expression of Aquaporin3 (AQP3), Aquaporin4 (AQP4), and Aquaporin8 (AQP8)

**Figure 5:**
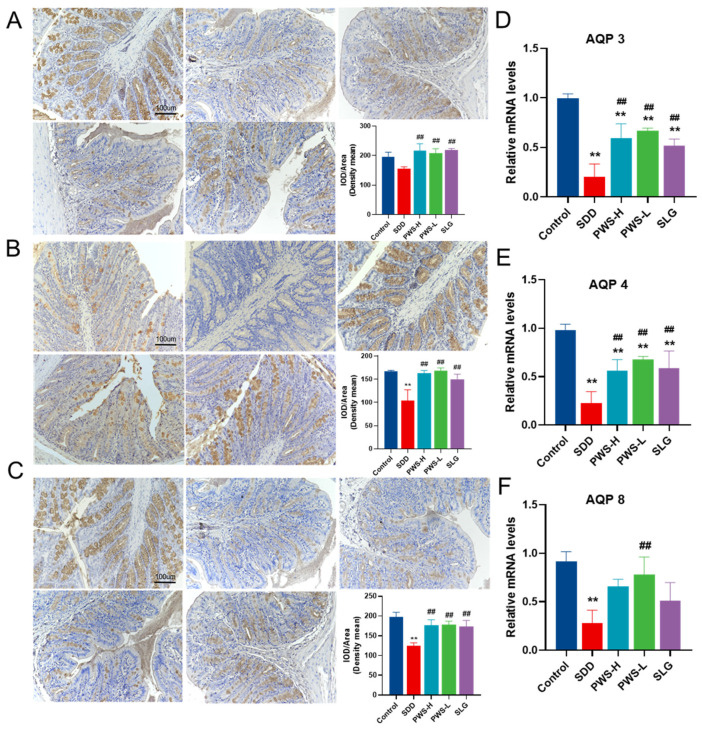
Effect of PWS on Aquaporins in SDD rats. The levels of AQP3 (**A**), AQP4 (**B**), and AQP8 (**C**) in the colon tissues. AQP3 (**D**), AQP4 (**E**), and AQP8 (**F**) expressions in the colon tissue measured by RT-qPCR. Control: control group; SDD: spleen-deficiency diarrhea group; PWS-H: Pingwei San high-dose group; PWS-L: Pingwei San low-dose group; SLG: Shenling Baizhu San group. The significance levels for *p*-values are indicated as follows: ** *p* < 0.01 vs. control group, ## *p* < 0.01 vs. DSS group.

Diarrhea is commonly associated with abnormal water metabolism in the intestine, which is regulated by AQP3, AQP4, and AQP8 proteins. In this study, we analyzed the expression of these proteins in colon tissue and found that their expression was significantly reduced in the SDD group (*p* < 0.05) but significantly increased in the PWS and SLG groups (*p* < 0.01) (Figure 5A–C). These findings were confirmed by qPCR results (Figure 5D–F).

### 3.6. PWS Relieved SDD by Maintaining Goblet Cell Function and Restoring the Intestinal Mucus Barrier

**Figure 6:**
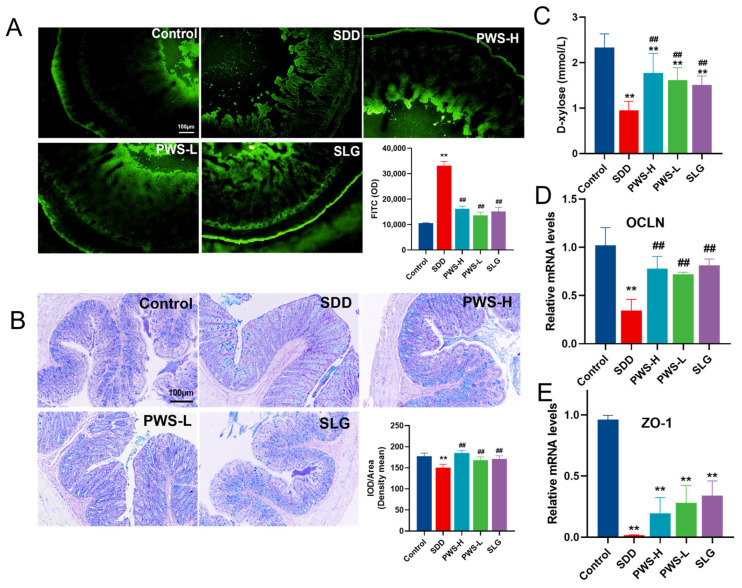
(**A**) Effect of PWS on the permeability of FITC-dextran. (**B**) AB-PAS images. (**C**) The effect of PWS on D-xylose concentration in SDD piglets. (**D**) The expression of *Ocln* in the colon tissue. (**E**) The expression of *Zo*-*1* in the colon tissue. Control: control group; SDD: spleen-deficiency diarrhea group; PWS-H: Pingwei San high-dose group; PWS-L: Pingwei San low-dose group; SLG: Shenling Baizhu San group.The significance levels for *p*-values are indicated as follows: ** *p* < 0.01 vs. control group, ## *p* < 0.01 vs. DSS group.

Regarding mucosal permeability, we evaluated the FD4 index, which reflects gut barrier function. Results showed that PWS reduced mucosal permeability, as evidenced by decreased fluorescence permeability of FITC-dextran and lower FD4 levels in serum (*p* < 0.01) (Figure 6A). The mucosal barrier of the intestine serves as the primary defense mechanism for safeguarding the intestinal epithelium. SDD reduced the number of goblet cells, but PWS and SLG attenuated this depletion (Figure 6B). Additionally, SDD rats had lower levels of D-xylose in their plasma, while rats given PWS had higher levels (*p* < 0.01) (Figure 6C). Furthermore, PWS upregulated the expression of *Ocln* and *Zo-1*, which were downregulated in the SDD group (Figure 6D,E). These results indicate that PWS protects goblet cell function and restores the mucus barrier in chronic colitis.

## 4. Discussion

PWS is a traditional Chinese medicine formula that has been used for gastrointestinal ailments for many years. Our results demonstrate that PWS mostly improved SDD by inhibiting the generation of inflammatory substances (TNF-α, IL-1β, and IL-6), enhancing the body’s antioxidant potential (TAOC, GSH-Px, and SOD), increasing the expression of aquaporins (AQP3, AQP4, and AQP8) and tight junction proteins (ZO-1 and Occludin), and stimulating mucin secretion from goblet cells in the colon. In addition, PWS significantly increased the abundance of *Prevotellaceae*, *Eubacterium_ruminantium_group*, and *Tuzzerella*, while reducing the abundance of *Ruminococcus* and *Frisingicoccus* in the feces of SDD rats. Through the LEfSe analysis, *Prevotella*, *Eubacterium_ruminantium_group*, and *Pantoea* were relatively enriched in the PWS group. Overall, the findings of this study indicate that PWS exerted a therapeutic effect on Rhubarb-induced SDD in rats by both protecting the intestinal barrier and modulating the imbalanced intestinal microbiota.

It is common to use rhubarb to induce a model of SDD [3,26]. Rhubarb anthraquinones (RA) are considered primary laxative elements, which fall under the category of anthranoid glycosides and encompass different types of anthraquinone derivatives, such as sennoside A, B, C, and D [27]. The primary laxative component is believed to be sennoside A (SA) [28]. In this study, the symptoms observed in the experimental group were consistent with those of SDD, indicating the successful establishment of the model. Prior research has demonstrated that diarrhea is typically accompanied by intestinal inflammation, severe dehydration, and intestinal barrier damage [29,30]. Decreased expression of tight junction (TJ) proteins such as ZO-1 and Occludin can weaken the intestinal epithelium integrity and cause colitis [31,32,33]. Our investigation demonstrated that the expression levels of ZO-1 and Occludin were diminished in the colonic tissue of rats with SDD. However, the administration of PWS notably ameliorated the expression of these proteins, thus enhancing the integrity of the intestinal epithelium and improving intestinal permeability. AQPs are transporters present in membranes that facilitate water absorption, secretion, and metabolism in the intestine. They may also regulate the tight junctions of the intestinal mucosa [34]. Our results indicate that rats with SDD exhibited a diminished mRNA expression of AQP3, AQP4, and AQP8 in the colon compared to the control group. Nevertheless, the use of PWS treatment heightened the expression of AQPs, resulting in water reabsorption within the colon and a decrease in fecal moisture. 

Intestinal inflammation and oxidative stress are widely recognized as key factors contributing to the development of mucosal barrier dysfunction, which can result in a range of gastrointestinal disorders [35,36]. Inflammation triggers the release of pro-inflammatory cytokines such as TNF-α, IL-1β, and IL-6 by immune cells, which can harm the intestinal epithelium and compromise the mucosal barrier [37]. Our investigation demonstrated that rats with SDD exhibited extensive inflammation infiltration in their colon tissue, coupled with tissue structure loss and reduced goblet cell count. However, PWS intervention mitigated the damage. Additionally, the results revealed a noteworthy elevation in the expression of proinflammatory cytokines (TNF-α, IL-1β, and IL-6) in rats with SDD. Nonetheless, the administration of PWS reduced the expression of these inflammatory cytokines. In addition to inflammation, oxidative stress is another important contributor to mucosal barrier dysfunction. In the gut, oxidative stress can lead to increased intestinal permeability, inflammation, and damage to the mucosal barrier [38]. GSH-Px, SOD, and TAOC are antioxidant enzymes that play important roles in protecting the body from oxidative stress. Previous studies have shown that animals with diarrhea often experience increased oxidative stress and decreased levels of GSH-Px, SOD, and TAOC in their bodies [39,40]. This suggests that oxidative stress may contribute to the development of diarrhea and that these antioxidant enzymes may be potential targets for the treatment of diarrhea. Our current data presented that PWS could significantly upregulate the levels of GSH-Px and TAOC and the activities of SOD in the colons of rats. Atractylenolide III, Atractylenolide II, Atractylenolide I, Magnolol, Honokiol, Hesperidin, Liquiritin, and Glycyrrhizic acid were identified as the key components responsible for the observed benefits of PWS through the UHPLC-MS/MS analysis. Several previous studies have highlighted the anti-inflammatory and antioxidant properties of these components [41,42,43,44]. A study has shown that Atractylenolide-1 was able to improve mucoprotein MUC2 and enhance the expression of tight junction proteins ZO-1 and Occludin in mice with colitis. This treatment also resulted in a significant decrease in pro-inflammatory cytokines TNF-α, IL-6, and IL-1β [45]. Magnolol and honokiol are bioactive compounds found in *Magnolia officinalis* Rehd. et Wils. that have been demonstrated to alleviate diarrhea and intestinal damage by regulating the calcium-activated potassium channels signaling pathway [46]. Taken together, PWS exerted a therapeutic effect on Rhubarb-induced SDD by repairing the intestinal barrier through anti-inflammation and anti-oxidation. 

The gut’s microbial community can positively impact host health by regulating nutrient metabolism, immune system modulation, and prevention of pathogenic colonization [47]. This investigation examined fecal bacterial changes in rats with diarrhea and their relationship. In rats with diarrhea, richness, evenness, and diversity were lower than in healthy rats, and the structure and membership of the bacterial community differed. Treatment with PWS restored the proportion of related bacterial genera in SDD rats to normal levels. Moreover, PWS significantly increased the abundance of *Prevotellaceae*, *Eubacterium_ruminantium_group*, and *Tuzzerella*, while reducing the abundance of *Ruminococcus* and *Frisingicoccus* in the feces of SDD rats. Through the LEfSe analysis, *Prevotella*, *Eubacterium_ruminantium_group*, and *Pantoea* were relatively enriched in the PWS group. *Prevotellaceae* is a crucial intestinal cornerstone bacterial genus that is closely associated with intestinal health [48]. The presence of *Prevotellaceae* in the gut can prevent the M1-like polarization of colonic macrophages and the production of proinflammatory factors, such as IL-1β and TNF-α [49]. *Eubacterium* can increase the bioavailability of butyrate to colonic epithelial cells [50]. Additionally, some studies have suggested that short-chain fatty acids (SCFAs) can exhibit anti-inflammatory effects by inhibiting pro-inflammatory cytokines, such as IFN-γ, IL-1β, IL-6, IL-8, and TNF-α, while increasing the expression of anti-inflammatory cytokines, such as IL-10 and TGF-β [51]. The bioactive compounds Liquiritin and Glycyrrhizic acid found in Shaoyao-Gancao-Tang has been demonstrated to decrease the abundance of bacteria of the genera *Ruminococcus* [52]. This finding is consistent with our study. Taken together, PWS exerted a therapeutic effect on Rhubarb-induced SDD by balancing the intestinal flora.

## 5. Conclusions

PWS significantly increased body weight, decreased fecal water content, and reduced inflammatory cell infiltration in the colon. It also alleviated colitis and promoted the expression of AQPs and tight junction markers, inhibiting the loss of colonic cup cells in rats with SDD induced by Rhubarb. In addition, PWS significantly increased the abundance of *Prevotellaceae*, *Eubacterium_ruminantium_group*, and *Tuzzerella*, while reducing the abundance of *Ruminococcus* and *Frisingicoccus* in the feces of SDD rats. Through the LEfSe analysis, *Prevotella*, *Eubacterium_ruminantium_group*, and *Pantoea* were relatively enriched in the PWS group. Overall, the findings of this study indicate that PWS exerted a therapeutic effect on Rhubarb-induced SDD in rats by both protecting the intestinal barrier and modulating the imbalanced intestinal microbiota. The exact mechanisms underlying the beneficial effects of PWS on SDD and its influence on the composition of gut microbiota remain unclear. Further research is needed to gain a better understanding of the underlying mechanisms that account for the therapeutic benefits of PWS treatment.

## Figures and Tables

**Table 1:** The gradient elution procedure.

Time	Flow Rate (mL/min)	%A	%B
0	0.3	100	0
10	0.3	70	30
25	0.3	60	40
30	0.3	50	50
40	0.3	30	70
45	0.3	0	100
60	0.3	0	100
60.5	0.3	100	0
70	0.3	100	0

**Table 2:** Primers and probes for qPCR.

Gene	Primer Sequence (5′–3′)	Gene Accession Number
*Zo-1*	F: CCATCTTTGGACCGATTGCTG R: TAATGCCCGAGCTCCGATG	NM_001106266.1
*Ocln*	F: GTCTTGGGAGCCTTGACATCTTG R: GCATTGGTCGAACGTGCATC	NM_031329.3
*Aqp3*	F: CCCAATGGCACAGCTGGTA R: GTCAACAATGGCCAGCACAC	NM_031703.2
*Aqp4*	F: CCATGGAACAACGCCAACTG R: CAGTGTATGGACCACCTCGAAAC	NM_001142366.2
*Aqp8*	F: GGCCTCAAGACCATGCTGCTA R: ACCTGCTCCTGCTCCTGGACTA	NM_019158.2
Gapdh	F: GCAAGTTCAACGGCACAG R: GCCAGTAGACTCCACGACAT	NM_017008.4

**Table 3:** Chemical composition of PWS.

Constituents	RT (min)	Calc.MW	Formula	Group Area	Reference Ion
Magnolol	38.606	266.1306	C18 H18 O2	1.3 × 10^10^	[M − H] − 1
Hesperidin	22.517	610.18966	C28 H34 O15	1.16 × 10^10^	[M − H] − 1
Liquiritin	21.484	418.12654	C21 H22 O9	5.79 × 10^9^	[M − H] − 1
Honokiol	36.539	266.1306	C18 H18 O2	7.3 × 10^8^	[M − H] − 1
Glycyrrhizic acid	31.424	822.40331	C42 H62 O16	1.22 × 10^8^	[M + H] + 1
Atractylenolide III	33.455	248.14131	C15 H20 O3	1.05 × 10^8^	[M + H] + 1
Atractylenolide II	37.76	232.14646	C15 H20 O2	85,619,188	[M + H] + 1
Atractylenolide I	40.772	230.1308	C15 H18 O2	47,974,522	[M + H] + 1

## Data Availability

The data presented in this study are available upon request from the corresponding author.

## Supplementary Materials

The following supporting information can be downloaded at: https://www.mdpi.com/article/10.3390/antiox12051122/s1, Table S1: Chemical composition of PWS.
