# Supplementary material for: Pingwei San Ameliorates Spleen Deficiency-Induced Diarrhea through Intestinal Barrier Protection and Gut Microbiota Modulation
Source: Antioxidants (Basel). 2023 May 19;12(5):1122. doi: 10.3390/antiox12051122 (PMC10215682; doi:10.3390/antiox12051122)
Supplement: Supplementary file 1 [file antioxidants-12-01122-s001.zip › antioxidants-2388331-supplementary.pdf]

antioxidants

Table S1. Chemical composition of PWS.

Name	RT [min]	Calc. MW	Formula	Group ArX10a: PWS	Reference Ion
DL-Stachydrine	1.528	143.0948	C7 H13 N O2	2.24×10^{10}	[M+H]+1
DL-Arginine	1.363	174.1118	C6 H14 N4 O2	2.12×10^{10}	[M+H]+1
(+)-Magnoflorine	20.083	341.16274	C20 H23 N O4	1.88×10^{10}	[M+H]+1
Proline	1.529	115.06344	C5 H9 N O2	1.8×10^{10}	[M+H]+1
Isoforsythiaside	20.151	624.2055	C29 H36 O15	1.67×10^{10}	[M-H]-1
N-(2,4-Dimethylphenyl formamide	2.683	149.08425	C9 H11 N O	1.39×10^{10}	[M+H]+1
Magnolol	38.606	266.1306	C18 H18 O2	1.3×10^{10}	[M-H]-1
Hesperidin	22.517	610.18966	C28 H34 O15	1.16×10^{10}	[M-H]-1
Diammonium glycyrrhizinate	29.737	822.40405	C42 H62 O16	9.81×10^{9}	[M-H]-1
Naringin	21.976	580.17936	C27 H32 O14	8.99×10^{9}	[M-H]-1
L-(-)-Malic acid	1.565	134.02151	C4 H6 O5	8.35×10^{9}	[M-H]-1
Purpureaside C	19.215	786.25855	C35 H46 O20	8.13×10^{9}	[M-H]-1
Choline	1.398	103.09974	C5 H13 N O	8.03×10^{9}	[M+H]+1
Citric acid	1.606	192.02711	C6 H8 O7	7.72×10^{9}	[M-H]-1
D-(-)-Quinic acid	1.475	192.06343	C7 H12 O6	6.1×10^{9}	[M-H]-1
Liquiritin	21.484	418.12654	C21 H22 O9	5.79×10^{9}	[M-H]-1
Nobiletin	32.114	402.13154	C21 H22 O8	5.7×10^{9}	[M+H]+1
Gluconic acid	1.407	196.05824	C6 H12 O7	5.52×10^{9}	[M-H]-1
Liguiritigenin-7-O-β-D-apiosyl-4'-O-β-D-glucoside	21.296	550.16877	C26 H30 O13	4.96×10^{9}	[M-H]-1
α,α-Trehalose	1.532	342.11618	C12 H22 O11	4.55×10^{9}	[M-H]-1
Isoliquiritigenin	21.489	256.07357	C15 H12 O4	4.42×10^{9}	[M+H]+1
Apomorphine	21.554	267.12587	C17 H17 N O2	3.34×10^{9}	[M+H]+1
Forsythoside E	17.429	462.1738	C20 H30 O12	3.16×10^{9}	[M-H]-1
Maltopentaose	5.179	828.27497	C30 H52 O26	3.02×10^{9}	[M-H]-1
Hesperetin	22.516	302.07867	C16 H14 O6	2.86×10^{9}	[M+H]+1
Pipecolic acid	1.555	129.07916	C6 H11 N O2	2.86×10^{9}	[M+H]+1
4-Oxoproline	3.094	129.04267	C5 H7 N O3	2.75×10^{9}	[M-H]-1
D-Raffinose	2.229	504.16919	C18 H32 O16	2.75×10^{9}	[M+FA-H]-1
Inositol	1.431	180.06343	C6 H12 O6	2.57×10^{9}	[M-H]-1
Melittoside	18.893	524.17433	C21 H32 O15	2.51×10^{9}	[M-H]-1
4-Vinylphenol	17.845	120.05751	C8 H8 O	2.49×10^{9}	[M+FA-H]-1

(15Z)-9,12,13-Trihydroxy-15-octa-decenoic acid	28.222	330.24058	C18 H34 O5	2.44×10⁹	[M-H]-1
Asparagine	1.35	132.05355	C4 H8 N2 O3	2.31×10⁹	[M-H]-1
Adenosine	8.613	267.09691	C10 H13 N5 O4	2.1×10⁹	[M+H]+1
Stachyose	3.305	666.22232	C24 H42 O21	2.04×10⁹	[M+FA-H]-1
Didymin	24.7	594.19495	C28 H34 O14	2.04×10⁹	[M+H]+1
BMK methyl glycidate	18.616	192.07869	C11 H12 O3	1.96×10⁹	[M+H]+1
trans-3-Indoleacrylic acid	17.008	187.06333	C11 H9 N O2	1.96×10⁹	[M+H]+1
Tangeritin	34.001	372.12099	C20 H20 O7	1.93×10⁹	[M+H]+1
Skimmin	20.162	324.08452	C15 H16 O8	1.88×10⁹	[M+H]+1
Ononin	23.737	430.12658	C22 H22 O9	1.84×10⁹	[M+H]+1
L-Phenylalanine	8.781	165.07914	C9 H11 N O2	1.57×10⁹	[M+H]+1
Vicenin II	19.547	594.15891	C27 H30 O15	1.56×10⁹	[M-H]-1
Adenine	2.548	135.05462	C5 H5 N5	1.47×10⁹	[M+H]+1
Trigonelline	1.505	137.04785	C7 H7 N O2	1.33×10⁹	[M+H]+1
L-Tryptophan	17.007	204.08987	C11 H12 N2 O2	1.31×10⁹	[M+H]+1
Glabrolide	27.14	468.32414	C30 H44 O4	1.3×10⁹	[M+H]+1
Naringenin	21.975	272.06829	C15 H12 O5	1.3×10⁹	[M+H]+1
Isosinensetin	28.871	372.12084	C20 H20 O7	1.28×10⁹	[M+H]+1
Methoxyphenamine	6.564	179.13114	C11 H17 N O	1.16×10⁹	[M+H]+1
Synephrine	2.681	167.09479	C9 H13 N O2	1.14×10⁹	[M+H]+1
Sinensetin	30.436	372.12084	C20 H20 O7	1.07×10⁹	[M+H]+1
3,4,5-Trimethoxyphenyl β-D-gluco-pyranoside	18.84	346.12656	C15 H22 O9	1.04×10⁹	[M+FA-H]-1
N-Acetyl-L-phenylalanine	20.783	207.08957	C11 H13 N O3	9.79×10⁸	[M-H]-1
Cinnamaldehyde	18.611	132.05751	C9 H8 O	9.66×10⁸	[M+H]+1
Isoliquiritin	23.729	418.12646	C21 H22 O9	9.4×10⁸	[M-H]-1
Plantamajoside	19.884	640.20069	C29 H36 O16	9.39×10⁸	[M-H]-1
β-D-Glucopyranuronic acid	1.41	194.04262	C6 H10 O7	9.1×10⁸	[M-H]-1
7-Methoxycoumarin	18.501	176.04738	C10 H8 O3	8.47×10⁸	[M+H]+1
Guanosine	9.246	283.09178	C10 H13 N5 O5	8.23×10⁸	[M-H]-1
Rutin	21.203	610.15365	C27 H30 O16	7.79×10⁸	[M-H]-1
Caffeic acid	19.431	180.04236	C9 H8 O4	7.76×10⁸	[M-H]-1
Fumaric acid	1.565	116.01087	C4 H4 O4	7.53×10⁸	[M-H]-1
Honokiol	36.539	266.1306	C18 H18 O2	7.3×10⁸	[M-H]-1
6-Demethoxytangeretin	30.56	342.11046	C19 H18 O6	7.19×10⁸	[M+H]+1

Chlorogenic acid	17.342	354.09513	C16 H18 O9	6.75×10⁸	[M-H]-1
L-Aspartic acid	1.361	133.0375	C4 H7 N O4	6.74×10⁸	[M-H]-1
Azelaic acid	23.03	188.10493	C9 H16 O4	6.58×10⁸	[M-H]-1
α-Cyperone	27.173	218.16704	C15 H22 O	6.54×10⁸	[M+H]+1
Guanine	9.257	151.04955	C5 H5 N5 O	6.44×10⁸	[M+H]+1
Curcumol	27.173	236.17757	C15 H24 O2	6.28×10⁸	[M+H]+1
Isosakuranetin	24.703	286.08381	C16 H14 O5	6.06×10⁸	[M+H]+1
Protocatechualdehyde	17.485	138.03168	C7 H6 O3	6.03×10⁸	[M-H]-1
2-Furoic acid	1.62	112.01601	C5 H4 O3	5.41×10⁸	[M-H]-1
Neochlorogenic acid	18.758	354.09487	C16 H18 O9	5.4×10⁸	[M+H]+1
Liquiritigenin	24.961	256.07359	C15 H12 O4	5.3×10⁸	[M-H]-1
Calceolarioside B	20.545	478.1476	C23 H26 O11	5.27×10⁸	[M-H]-1
Bioside	18.503	462.17377	C20 H30 O12	5.25×10⁸	[M-H]-1
18 β-Glycyrrhetintic Acid	29.739	470.33965	C30 H46 O4	5.19×10⁸	[M+H]+1
Docosanamide	55.371	339.35007	C22 H45 N O	5.06×10⁸	[M+H]+1
Guanosine-3',5'-cyclic monophosphate	6.256	345.04753	C10 H12 N5 O7 P	4.88×10⁸	[M-H]-1
Prunin	21.989	434.12132	C21 H22 O10	4.86×10⁸	[M+H]+1
Eriocitrin	21.014	596.17457	C27 H32 O15	4.85×10⁸	[M-H]-1
Poliumoside	19.793	770.26393	C35 H46 O19	4.81×10⁸	[M-H]-1
Morphine	19.455	285.13652	C17 H19 N O3	4.65×10⁸	[M+H]+1
Corchorifatty acid F	27.047	328.22493	C18 H32 O5	4.51×10⁸	[M-H]-1
Isoalantolactone	17.804	232.14639	C15 H20 O2	4.34×10⁸	[M+H]+1
Narirutin	19.801	580.17949	C27 H32 O14	4.2×10⁸	[M+H]+1
Nicotinamide	3.272	122.04809	C6 H6 N2 O	4.03×10⁸	[M+H]+1
Limonin	21.2	470.19417	C26 H30 O8	4.01×10⁸	[M+H]+1
Rhoifolin	22.112	578.16411	C27 H30 O14	3.86×10⁸	[M+FA-H]-1
Ferulic acid	19.22	194.05795	C10 H10 O4	3.41×10⁸	[M+H]+1
4-Indolecarbaldehyde	17.008	145.05278	C9 H7 N O	3.32×10⁸	[M+H]+1
2-Methylbenzoic acid	19.431	136.0525	C8 H8 O2	3.28×10⁸	[M-H]-1
Succinic acid	3.816	118.02666	C4 H6 O4	3.28×10⁸	[M-H]-1
Argininosuccinic acid	2.537	290.12281	C10 H18 N4 O6	3.2×10⁸	[M+H]+1
Eleutheroside E	20.305	742.2688	C34 H46 O18	3.2×10⁸	[M+FA-H]-1
4-Guanidinobutyric acid	2.419	145.0853	C5 H11 N3 O2	3.07×10⁸	[M+H]+1
Isorhamnetin-3-O-rutinoside	22.119	624.1693	C28 H32 O16	2.89×10⁸	[M+H]+1
Formononetin	23.736	268.07346	C16 H12 O4	2.87×10⁸	[M+H]+1

9-Oxo-ODE	28.219	294.21959	C18 H30 O3	2.87×10^8	[M+H]+1
Germacrone	25.891	218.16704	C15 H22 O	2.86×10^8	[M+H]+1
2-Isopropylmalic acid	17.872	176.06846	C7 H12 O5	2.84×10^8	[M-H]-1
Glutaric anhydride	1.435	114.03167	C5 H6 O3	2.76×10^8	[M-H]-1
L-Histidine	1.366	155.06964	C6 H9 N3 O2	2.75×10^8	[M+H]+1
Maleic acid	1.982	116.01087	C4 H4 O4	2.73×10^8	[M-H]-1
6-Methylquinoline	17.008	143.07353	C10 H9 N	2.73×10^8	[M+H]+1
2-Hydroxy-4-methoxybenzaldehyde	18.878	152.04736	C8 H8 O3	2.67×10^8	[M+H]+1
Benzoic acid	19.513	122.03677	C7 H6 O2	2.61×10^8	[M-H]-1
Uridine	5.093	244.06958	C9 H12 N2 O6	2.61×10^8	[M-H]-1
3-Hydroxybenzoic acid	23.022	138.03168	C7 H6 O3	2.51×10^8	[M-H]-1
Acetophenone	5.15	120.05756	C8 H8 O	2.48×10^8	[M+H]+1
5-Amino-3-(4-methoxyphenyl)-5-oxopentanoic acid	7.24	237.10013	C12 H15 N O4	2.34×10^8	[M+H]+1
Quillaic acid	28.556	486.33434	C30 H46 O5	2.26×10^8	[M+H]+1
Vicenin III	20.246	564.14823	C26 H28 O14	2.25×10^8	[M+H]+1
1,2,3,4-Tetramethyl-1,3-cyclopentadiene	30.296	122.10958	C9 H14	2.25×10^8	[M+H]+1
Lariciresinol 4-O-glucoside	21.286	522.21035	C26 H34 O11	2.17×10^8	[M+FA-H]-1
2,3-Dihydroxybenzoic acid	14.75	154.02669	C7 H6 O4	2.1×10^8	[M-H]-1
5-O-Demethylnobiletin	35.702	388.11595	C20 H20 O8	2.06×10^8	[M+H]+1
Isosakuranin	24.704	448.13674	C22 H24 O10	2.02×10^8	[M+H]+1
Anabasine	6.484	162.11577	C10 H14 N2	1.93×10^8	[M+H]+1
trans-Aconitic acid	1.614	174.01639	C6 H6 O6	1.92×10^8	[M-H]-1
Kojic acid	15.643	142.0268	C6 H6 O4	1.77×10^8	[M+H]+1
Curdione	25.974	236.17757	C15 H24 O2	1.75×10^8	[M+H]+1
D-Glucosamine	1.417	179.07955	C6 H13 N O5	1.7×10^8	[M+H]+1
(±)-Abscisic acid	24.796	264.1362	C15 H20 O4	1.68×10^8	[M-H]-1
2'-Deoxyadenosine	9.988	251.10192	C10 H13 N5 O3	1.64×10^8	[M+H]+1
Kaempferol-3-O-rutinoside	21.977	594.15891	C27 H30 O15	1.58×10^8	[M-H]-1
Phenibut	5.115	179.09473	C10 H13 N O2	1.57×10^8	[M+H]+1
Methyl 4-hydroxy-3-methoxycinnamate	23.11	208.0737	C11 H12 O4	1.53×10^8	[M+H]+1
Oleamide	47.093	281.27202	C18 H35 N O	1.51×10^8	[M+H]+1
Boldine	20.698	327.14696	C19 H21 N O4	1.44×10^8	[M+H]+1

Icaritin	33.038	368.12615	C21 H20 O6	1.43×10^8	[M-H]-1
Sarracenin	9.026	226.08399	C11 H14 O5	1.43×10^8	[M+H]+1
(+)-Syringaresinol	22.219	418.16293	C22 H26 O8	1.42×10^8	[M-H]-1
Quercetin	21.206	302.04253	C15 H10 O7	1.4×10^8	[M+H]+1
L-Tyrosine	1.678	181.07413	C9 H11 N O3	1.37×10^8	[M+H]+1
Adenosine 5'-monophosphate	2.806	347.06324	C10 H14 N5 O7 P	1.36×10^8	[M+H]+1
4,5-Dicaffeoylquinic acid	22.806	516.12701	C25 H24 O12	1.3×10^8	[M-H]-1
Cytosine	2.47	111.04332	C4 H5 N3 O	1.29×10^8	[M+H]+1
1,3-Dicaffeoylquinic acid	22.348	516.12703	C25 H24 O12	1.28×10^8	[M-H]-1
o-Veratraldehyde	17.134	166.0631	C9 H10 O3	1.27×10^8	[M+H]+1
Parthenolide	26.674	248.14131	C15 H20 O3	1.26×10^8	[M+H]+1
(-)-Caryophyllene oxide	24.637	220.1828	C15 H24 O	1.24×10^8	[M+H]+1
Uracil	5.091	112.0273	C4 H4 N2 O2	1.23×10^8	[M+H]+1
Glycyrrhizic acid	31.424	822.40331	C42 H62 O16	1.22×10^8	[M+H]+1
Adenosine 3'5'-cyclic monophosphate	5.239	329.05266	C10 H12 N5 O6 P	1.21×10^8	[M+H]+1
5'-S-Methyl-5'-thioadenosine	17.716	297.08965	C11 H15 N5 O3 S	1.21×10^8	[M+H]+1
Methyl cinnamate	18.23	162.06809	C10 H10 O2	1.18×10^8	[M+H]+1
Acetylarginine	2.502	216.12264	C8 H16 N4 O3	1.18×10^8	[M+H]+1
Wilforlide A	25.054	454.34462	C30 H46 O3	1.17×10^8	[M+H]+1
Codeine	21.278	299.15212	C18 H21 N O3	1.11×10^8	[M+H]+1
Paratocarpin G	39.891	424.1888	C25 H28 O6	1.08×10^8	[M-H]-1
4-Aminophenol	3.007	109.05285	C6 H7 N O	1.07×10^8	[M+H]+1
Isomeranzin	23.953	260.10488	C15 H16 O4	1.07×10^8	[M+H]+1
Neodiosmin	22.309	608.17468	C28 H32 O15	1.06×10^8	[M+H]+1
Atractylenolide III	33.455	248.14131	C15 H20 O3	1.05×10^8	[M+H]+1
Methyl hesperidin	23.618	624.20596	C29 H36 O15	1.04×10^8	[M-H]-1
4-Methoxysalicylic acid	16.807	168.04225	C8 H8 O4	1.04×10^8	[M-H]-1
Nabumetone	24.804	228.11507	C15 H16 O2	1.03×10^8	[M+H]+1
Apigenin 7-O-(2G-rhamnosyl)gentiobioside	19.851	740.21666	C33 H40 O19	1.02×10^8	[M+H]+1
2-Hydroxyacetophenone	16.635	136.05252	C8 H8 O2	1.01×10^8	[M+H]+1
1-Caffeoylquinic acid	16.436	354.09512	C16 H18 O9	9.93×10^7	[M-H]-1
4-(3,4-Dihydroxyphenyl)-6,7-dihydroxy-2-naphthoic acid	26.22	312.06357	C17 H12 O6	9.9×10^7	[M-H]-1
N-Acetyl-DL-tryptophan	21.682	246.10045	C13 H14 N2 O3	9.72×10^7	[M-H]-1

3-Methoxy-5,7,3',4'-tetrahydroxy-flavone	22.109	316.05831	C16 H12 O7	9.56×10^{7}	[M+H]+1
3-tert-Butyladipic acid	24.974	202.12057	C10 H18 O4	9.22×10^{7}	[M-H]-1
5,7,3',4'-Tetrahydroxy-6,8-diprenyli-soflavone	41.208	422.17326	C25 H26 O6	9.21×10^{7}	[M+H]+1
Catechol	14.747	110.03672	C6 H6 O2	8.81×10^{7}	[M-H]-1
Demethylcoclaurine hydrochloride	17.977	271.12074	C16 H17 N O3	8.78×10^{7}	[M+H]+1
Salicylic acid	17.952	138.03168	C7 H6 O3	8.78×10^{7}	[M-H]-1
Scopoletin	21.744	192.04239	C10 H8 O4	8.75×10^{7}	[M+H]+1
3-Methoxybenzaldehyde	20.548	136.05252	C8 H8 O2	8.74×10^{7}	[M+H]+1
Atractylenolide II	37.76	232.14646	C15 H20 O2	8.56×10^{7}	[M+H]+1
Sinapic acid	21.783	224.06851	C11 H12 O5	8.55×10^{7}	[M-H]-1
19-Nortestosterone	27.07	274.19328	C18 H26 O2	8.48×10^{7}	[M+H]+1
Scoparone	23.959	206.05795	C11 H10 O4	8.48×10^{7}	[M+H]+1
Sedanolide	28.24	194.13082	C12 H18 O2	8.38×10^{7}	[M+H]+1
1,5-Naphthalenediamine	16.998	158.08441	C10 H10 N2	7.94×10^{7}	[M+H]+1
Leucylproline	17.42	228.14746	C11 H20 N2 O3	7.93×10^{7}	[M+H]+1
7-Hydroxycoumarin	21.583	162.03172	C9 H6 O3	7.81×10^{7}	[M-H]-1
3-Coumaric acid	21.093	164.04743	C9 H8 O3	7.64×10^{7}	[M-H]-1
α-Lactose	2.232	342.1165	C12 H22 O11	7.5×10^{7}	[M+H]+1
Calycosin-7-O-β-D-glucoside	21.238	446.12132	C22 H22 O10	7.46×10^{7}	[M+H]+1
Dibutyl phthalate	43.028	278.15192	C16 H22 O4	7.17×10^{7}	[M+H]+1
D-Saccharic acid	18.56	210.03752	C6 H10 O8	6.96×10^{7}	[M-H]-1
9S,13R-12-Oxophytodienoic acid	27.045	292.20366	C18 H28 O3	6.88×10^{7}	[M+H]+1
Cytidine	2.467	243.08568	C9 H13 N3 O5	6.86×10^{7}	[M+H]+1
Padmatin	19.854	318.07393	C16 H14 O7	6.8×10^{7}	[M+H]+1
Phlinoside A	21.473	786.25859	C35 H46 O20	6.65×10^{7}	[M-H]-1
DL-Lysine	1.217	146.10562	C6 H14 N2 O2	6.57×10^{7}	[M+H]+1
Guanosine monophosphate (GMP)	3.075	363.05817	C10 H14 N5 O8 P	6.42×10^{7}	[M-H]-1
Syringic acid	17.672	198.05282	C9 H10 O5	6.41×10^{7}	[M-H]-1
Skatole	17.002	131.07352	C9 H9 N	6.38×10^{7}	[M+H]+1
Artemetin	32.612	388.11597	C20 H20 O8	6.35×10^{7}	[M+H]+1
Coumarin	21.03	146.03676	C9 H6 O2	6.23×10^{7}	[M+H]+1
Penicillic acid	17.14	170.05795	C8 H10 O4	6.13×10^{7}	[M+H]+1
Ferulaldehyde	5.949	178.06316	C10 H10 O3	6.11×10^{7}	[M+H]+1
Tectorigenin	25.007	300.06324	C16 H12 O6	6.02×10^{7}	[M-H]-1

Dehydrodiisoeugenol	23.376	326.15166	C20 H22 O4	5.97×10^{7}	[M+H]+1
7-Methoxy-4-methylcoumarin	20.475	190.06311	C11 H10 O3	5.94×10^{7}	[M+H]+1
Riboflavin	19.65	376.13838	C17 H20 N4 O6	5.89×10^{7}	[M+H]+1
5,2'-Dihydroxy-6,7,8,6'-tetramethoxyflavone	32.419	374.10037	C19 H18 O8	5.83×10^{7}	[M+H]+1
Pinocembrin	24.496	256.07357	C15 H12 O4	5.79×10^{7}	[M+H]+1
Catechol	15.603	110.03672	C6 H6 O2	5.75×10^{7}	[M-H]-1
(2Z)-2-Octyl-2-pentenedioic acid	20.946	242.15188	C13 H22 O4	5.75×10^{7}	[M-H]-1
4',7-Dihydroxyflavanone	26.048	256.07356	C15 H12 O4	5.63×10^{7}	[M+H]+1
Matairesinol	21.718	358.14158	C20 H22 O6	5.52×10^{7}	[M+H]+1
4-Coumaric acid	23.588	164.04742	C9 H8 O3	5.46×10^{7}	[M-H]-1
Orientin	20.65	448.10083	C21 H20 O11	5.46×10^{7}	[M-H]-1
3,4-Dimethylbenzoic acid	19.156	150.06813	C9 H10 O2	5.37×10^{7}	[M+H]+1
Suberic acid	21.12	174.08911	C8 H14 O4	5.33×10^{7}	[M-H]-1
4-Acetyl-3-hydroxy-5-methylphenyl β-D-glucopyranoside	19.361	328.11592	C15 H20 O8	5.28×10^{7}	[M-H]-1
Pantothenic acid	16.8	219.11078	C9 H17 N O5	5.24×10^{7}	[M+H]+1
Eriodictyol	21.027	288.06346	C15 H12 O6	5.19×10^{7}	[M+H]+1
Atractylenolide I	40.772	230.1308	C15 H18 O2	4.79×10^{7}	[M+H]+1
Obacunone	23.897	454.19902	C26 H30 O7	4.73×10^{7}	[M+H]+1
3,4-Dihydroxyphenylethanol	15.867	154.063	C8 H10 O3	4.69×10^{7}	[M-H]-1
Uridine monophosphate (UMP)	2.23	324.03572	C9 H13 N2 O9 P	4.59×10^{7}	[M-H]-1
Quinic acid	16.434	192.06343	C7 H12 O6	4.4×10^{7}	[M-H]-1
Glabrone	38.64	336.09969	C20 H16 O5	4.35×10^{7}	[M+H]+1
Arglabin	29.111	246.1256	C15 H18 O3	4.35×10^{7}	[M+H]+1
Abscisic acid	23.965	264.13608	C15 H20 O4	4.31×10^{7}	[M-H]-1
α-Eleostearic acid	40.204	278.22469	C18 H30 O2	4.3×10^{7}	[M+H]+1
Pholedrine	7.212	165.11548	C10 H15 N O	4.29×10^{7}	[M+H]+1
1-Methyladenosine	16.219	281.11244	C11 H15 N5 O4	4.29×10^{7}	[M+H]+1
Medicarpin	25.402	270.08916	C16 H14 O4	4.2×107	[M+H]+1
Glycitin	23.368	446.12147	C22 H22 O10	4.18×10^{7}	[M+H]+1
(9Z,12Z)-6,8-Dihydroxy-9,12-octadecadienoic acid	35.763	312.23024	C18 H32 O4	3.79×10^{7}	[M-H]-1
Secoisolariciresinol	23.377	362.17326	C20 H26 O6	3.72×10^{7}	[M-H]-1
Citral	22.472	152.12018	C10 H16 O	3.68×10^{7}	[M+H]+1
Rosarin	22.62	428.16786	C20 H28 O10	3.65×10^{7}	[M-H]-1
Linderalactone	23.501	244.10993	C15 H16 O3	3.63×10^{7}	[M+H]+1
16α-Hydroxyestrone	27.474	286.15833	C18 H22 O3	3.6×10^{7}	[M+H]+1

Shikimic acid	2.154	174.05273	C7 H10 O5	3.51×10^{7}	[M-H]-1
(+/-)9,10-dihydroxy-12Z-octadecenoic acid	35.808	314.246	C18 H34 O4	3.43×10^{7}	[M-H]-1
2-Hydroxybenzyl alcohol	15.876	124.05242	C7 H8 O2	3.39×10^{7}	[M-H]-1
β-Asarone	26.02	208.10993	C12 H16 O3	3.38×10^{7}	[M+H]+1
β-Asarone	21.896	208.10993	C12 H16 O3	3.37×10^{7}	[M+H]+1
Manninotriose	5.195	504.16934	C18 H32 O16	3.29×10^{7}	[M-H]-1
Curcumenol	28.198	234.16207	C15 H22 O2	3.07×10^{7}	[M+H]+1
Genistein	27.246	270.05286	C15 H10 O5	2.88×10^{7}	[M-H]-1
Gardenin B	27.929	358.10524	C19 H18 O7	2.87×10^{7}	[M+H]+1
16-Hydroxyhexadecanoic acid	45.815	272.23527	C16 H32 O3	2.85×10^{7}	[M-H]-1
Calycosin	25.38	284.06847	C16 H12 O5	2.77×10^{7}	[M+H]+1
2',4'-Dihydroxydihydrochalcone	29.382	242.0943	C15 H14 O3	2.75×10^{7}	[M-H]-1
Jasmonic acid	29.849	210.12557	C12 H18 O3	2.74×10^{7}	[M+H]+1
Fraxinellone	23.491	232.10994	C14 H16 O3	2.69×10^{7}	[M+H]+1
(+/-)12(13)-DiHOME	35.393	314.24598	C18 H34 O4	2.68×10^{7}	[M-H]-1
Xanthosine	15.127	284.07564	C10 H12 N4 O6	2.61×10^{7}	[M-H]-1
2'-Deoxyadenosine-5'-monophosphate	4.779	331.06841	C10 H14 N5 O6 P	2.53×10^{7}	[M+H]+1
Isoferulic acid	24.234	194.05792	C10 H10 O4	2.51×10^{7}	[M-H]-1
Safrole	33.451	162.06816	C10 H10 O2	2.48×10^{7}	[M+H]+1
Methohexital	17.927	262.1317	C14 H18 N2 O3	2.45×10^{7}	[M+H]+1
Thymol	28.233	150.10454	C10 H14 O	2.43×10^{7}	[M+H]+1
β-Estradiol	29.172	272.1776	C18 H24 O2	2.38×10^{7}	[M+H]+1
Daidzein	24.307	254.05791	C15 H10 O4	2.36×10^{7}	[M+H]+1
Alisol C 23-acetate	27.024	528.34545	C32 H48 O6	2.15×10^{7}	[M+H]+1
Shogaol	32.04	276.17281	C17 H24 O3	2.13×10^{7}	[M+H]+1
Osajin	45.214	404.16271	C25 H24 O5	2.06×10^{7}	[M+H]+1
1-Hydroxycyclohexyl phenyl ketone	24.809	204.11508	C13 H16 O2	1.82×10^{7}	[M+H]+1
Xanthine	4.605	152.03351	C5 H4 N4 O2	1.74×10^{7}	[M-H]-1
Eupatilin	31.77	344.08951	C18 H16 O7	1.58×10^{7}	[M+H]+1
TDIQ	15.977	177.07901	C10 H11 N O2	1.58×10^{7}	[M+H]+1
4'-Methoxychalcone	24.292	238.09931	C16 H14 O2	1.36×10^{7}	[M+H]+1
Testosterone 17-Undecanoate	46.381	456.36061	C30 H48 O3	1.27×10^{7}	[M-H]-1
Racemosin	31.472	288.09975	C16 H16 O5	1.27×10^{7}	[M+H]+1
3-Allyl-2-hydroxybenzoic acid	30.191	178.06309	C10 H10 O3	1.25×10^{7}	[M-H]-1
Mulberrin	42.906	422.1732	C25 H26 O6	1.24×10^{7}	[M+H]+1

Ambrosic acid	27.751	264.13606	C15 H20 O4	1.23×10^{7}	[M-H]-1
3-Hydroxyfluorene	32.986	182.07335	C13 H10 O	1.2×10^{7}	[M+H]+1
Dodecanedioic acid	29.252	230.15176	C12 H22 O4	1.17×10^{7}	[M-H]-1
Hexadecanedioic acid	37.764	286.21452	C16 H30 O4	1.17×10^{7}	[M-H]-1
Indole-3-lactic acid	16.464	205.07405	C11 H11 N O3	1.06×10^{7}	[M+H]+1
9-Oxo-10(E),12(E)-octadecadienoic acid	41.767	294.21964	C18 H30 O3	1.04×10^{7}	[M+H]+1
Clareolide	30.111	250.19332	C16 H26 O2	1.01×10^{7}	[M+H]+1
Cyclohexyl phenyl ketone	39.367	188.12024	C13 H16 O	9.34×10^{6}	[M+H]+1
α-Linolenic acid	40.782	278.22469	C18 H30 O2	8.7×10^{6}	[M+H]+1
Biochanin A	32.996	284.06863	C16 H12 O5	8.58×10^{6}	[M+H]+1
Docosatrienoic acid	46.213	334.28722	C22 H38 O2	8.45×10^{6}	[M+H]+1
3,3',5,5'-Tetramethyldiphenoquinone	40.497	240.11507	C16 H16 O2	7.29×10^{6}	[M+H]+1
Roburic acid	51.106	440.36568	C30 H48 O2	6.82×10^{6}	[M+H]+1
Kanzakiflavone 2	34.813	298.04781	C16 H10 O6	6.05×10^{6}	[M+H]+1
Bavachinin	39.007	338.15221	C21 H22 O4	5.49×10^{6}	[M+H]+1
Ciliatin A	39.25	336.09972	C20 H16 O5	4.08×10^{6}	[M+H]+1
Oleoyl ethanolamide	46.115	325.29823	C20 H39 N O2	3.35×10^{6}	[M+H]+1
Polygalic acid	28.289	488.31404	C29 H44 O6	1.9×10^{6}	[M+H]+1
